# Understanding Patterns of Economic Insecurity for Post-Soviet Migrant Women in Europe

**DOI:** 10.3389/fsoc.2021.614713

**Published:** 2021-04-15

**Authors:** Irina Gewinner, Stefania Salvino

**Affiliations:** ^1^Institute of Education and Society, University of Luxembourg, Esch-sur-Alzette, Luxembourg; ^2^Department of Sociology, Faculty of Humanities, Leibniz University Hannover, Hanover, Germany; ^3^Department of Sociology and Political Science, University of Calabria, Cosenza, Italy

**Keywords:** strategies against economic insecurity, Russian-speaking migrant women, Germany, Italy, Spain

## Abstract

This study deals with meanings of economic insecurity for post-Soviet migrant women in Germany, Italy, and Spain, elaborating on its cultural underpinnings. Drawing upon several data sources, including interviews, observation, and online data, as well as judicial material, this study addresses the ways women from the former Soviet Union experience economic insecurity and which strategies they develop to cope. We consider women's age, social background, and level of education, analyzing their embeddedness into different life domains. We identify four patterns of coping with economic insecurity, linked to individual characteristics, cultural values and legal frame conditions in the countries under investigation, and provide implications for social mobility and conservative backlash in Europe.

## Introduction

In the context of studies on migration from the former Soviet Union (FSU) to Western Europe, previous research has predominantly discussed immigrants' vulnerability, reinforcing the image of settlers in precarious and insecure living conditions, seeking a better life in more prosperous European countries. Within this discourse, vulnerability has been primarily understood as absence of formal employment, and economic insecurity as lack of financial resources in the form of earnings, property, and savings (Young, 2001; Krishnan, 2010; Freedman, 2012; Harney, 2012). Studies developed an argument that lack of integration, the latter equated with a stable job, is caused by migrants' insufficient pursuit in recognition of their educational qualifications in receiving societies (Kogan, 2012) or by low transferability of skills to the labor markets of destination countries. This, in turn, often results in loss of individual social status and the need to start from a scratch in a new place.

Human capital explanations dominate the current discourse that foregrounds integration of migrants (European Commission, 2011; Phillimore, 2012; Koshulko and Kostjukova, 2017). Research within this tradition, criticized for its high normativity load (Anthias, 2013), suggests that migrants are unevenly equipped with skills, which is decisive for the labor market and their subsequent social integration. Individuals without competitive qualifications and language skills are exposed to economic insecurity in terms of unemployment or precarious positions. While this line of reasoning is convincing, it fails to explain why migrants from the FSU, who usually demonstrate relatively high levels of education, languidly undergo recognition procedure of their educational credentials or obtain new qualifications, and struggle with insecurity. This study explores to what extent women from the FSU face economic insecurity and how this relates to their cultural backgrounds. Understanding patterns of insecurity and coping strategies is especially relevant in the light of diversification of migration motives and immigrant policies in the EU, which no longer allow clear differentiation between groups of migrants and require flexible policy solutions. It is particularly important in terms of social cohesion in receiving societies, in line with sustainable development goals and equal opportunities for all.

Contributing to the discussion on post-socialist migration, this article investigates strategies of coping with economic insecurity in post-Soviet migrant women in Europe from a cultural perspective. It enriches the body of knowledge by providing a female gaze on migration and its aftermath, and reports on experiences of women, which often differ from the notion of young economically-driven men, assumed by policies. To provide a comprehensive picture, the study addresses women in Italy, Germany, and Spain, old and new immigration countries. It contributes to the discussion on cultural underpinnings of economic insecurity by exploring narratives of migration and (non-)work in women who were socialized in the FSU and migrated to Europe in 1990–2010. Each receiving country is associated with different types of migration. Italy represents low-skilled care migration (Scrinzi, 2004; Marchetti, 2013, 2017). Spain is associated with lifestyle and marriage migration (Roca and Umeneta, 2013). Germany, which—like Italy—registers more female than male migrants from post-socialist countries (Statistisches Bundesamt, 2020), is attractive for very diverse social groups ranging from highly-skilled (Gewinner, 2019, 2020) to usually less skilled ethnic Germans (Kogan, 2012).

The target group is of special interest and relevance for several reasons. First, female migration from the FSU to Europe, which developed a few years after the fall of the Berlin Wall, has assumed considerable weight over time, becoming a significant phenomenon both numerically and in terms of territorial rooting strategies (Salvino, 2018). Post-Soviet women from Russia and Ukraine outnumber men migrating to Germany and, together with women from other post-Soviet countries, constitute one of the largest migrant women groups there (Statistisches Bundesamt, 2020). In Italy and Spain, communities from the FSU are relatively large (Vianello et al., 2019). Second, there is almost no consistent analysis of the experiences of female migrants and the “female face” of migration from the former USSR, although female professionals constitute a considerable share in highly-skilled migration flows (Zaionchkovskaya, 2004), in particular among youth (Mosakova, 2017). Third, women are highly involved in both production and reproduction processes, which goes along with persistent gender inequalities in the post-Soviet space. Support of traditional family values and gender roles therefore coexists with women's relatively high levels of education and employment (Gewinner, 2019).

## Current State of Research on Female Migration From FSU

### Post-Soviet Female Immigrants in Germany, Italy, and Spain

Migration of post-Soviet women to Western and Southern Europe was induced by specific pull factors emerging from receiving contexts (work, care, education, or private reasons), as well as by push factors within the sending contexts (dissolution of the USSR and loss of social and economic guarantees). Contemporary women's transnational migrations are triggered by the fall of state socialism and by globalization, which sometimes produces the effect known as “contradictory class mobility” (Parreñas, 2015).

Previous research on post-Soviet immigration has concentrated on single groups of migrants, mostly defined by a motive of migration, or single countries. For instance, for Germany, studies compared ethnic Germans with Jewish immigrants in terms of labor market integration (Burkert, 2011; Haberfeld et al., 2011; Kogan, 2012; Remennick, 2012; Panagiotidis, 2017b), with noteworthy exceptions focusing on highly-skilled female professionals (Gewinner, 2019; Antoshchuk and Gewinner, 2020). In Italy, research has addressed post-Soviet migrants with particular reference to Ukrainian and Moldovan women, who constitute the majority of post-Soviet migrants. Studies associate them with either low-skilled care work and a boom of domestic regime (Torre, 2008; Hrycak, 2011; Solari, 2011, 2018; Boccagni and Ambrosini, 2012; Fedyuk, 2012, 2016; Vianello, 2014, 2016; Di Bartolomeo et al., 2015) or love relationships and mixed marriages (Salvino, 2014, 2017; Cvajner, 2018, 2019). In Spain, scarce investigations on post-Soviet migrants found that the latter fill such secondary labor market niches as domestic or care work and construction (León, 2010; Aretxabala and Gorodetska, 2012; Domínguez-Mujica et al., 2014; Hobson et al., 2015; Gea-Sánchez et al., 2017; Vianello et al., 2019). Two studies foreground post-Soviet women's marriage migration (Roca and Umeneta, 2013; Girona et al., 2017).

Due to their social composition, migrant women from the FSU constitute a multifaceted social group in Europe and elsewhere, which can hardly be studied under one particular research frame alone. Unlike other groups that make up the bulks of certain migration flows (e.g., labor or marriage migrants), post-Soviet immigrants vary widely in their migration motives and patterns. Especially women differ along the axes of social inequality, such as socioeconomic background, education, qualifications, and work experience, place of origin, but also foreign language proficiency and socialization.

Although post-Soviet women in Europe represent a very diverse migrant group, especially with regard to the vast geography of the FSU, it can be assumed that they have a common cultural background (Gewinner, 2020; Salvino, 2020a,b). This cultural context incorporates (post-)Soviet collective memory, Russian language proficiency, and certain cultural values, such as a strong work ethic and iron discipline (Ashwin, 2000), as well as conformity and security, both standing for “conservation” (Magun and Rudnev, 2012). Previous research has also demonstrated the importance of power, wealth, social recognition, and personal success for post-Soviet people, all standing in contrast to social equality, concern for the welfare of other people, and the environment (Magun and Rudnev, 2012; Maslova and King, 2020).

Despite relatively high levels of education, migrant women from the FSU tend to support traditional gender role models. Solid proportions of post-Soviet migrant women in Europe consider marriage a route to success in life or a traditionally rooted necessity, forming the woman's role (Salvino, 2014, 2017; Gewinner, 2019, 2020; Antoshchuk and Gewinner, 2020). These orientations have historical roots, being valid throughout the Soviet period regardless of proclaimed gender equality, and reinforced by post-socialist policies with the support of the Orthodox church as early as 2008 (Stoeckl, 2016). Previous research has found that cultural values, transmitted during socialization, dominated the attitudes and opinions of post-Soviet migrants even after their change of living environment (Hedegaard and Bekhuis, 2018; Gewinner, 2020).

### Coping With Economic Insecurity in Host Countries

Integration of newcomers into receiving societies is key to research to date (Kogan, 2011, Anthias et al., 2012; Aretxabala and Gorodetska, 2012; Rodríguez-Planas and Nollenberger, 2014; Panagiotidis, 2017a; Ryan, 2018). Although it yielded criticism for heating securitization discussion and strengthening the division between natives and strangers (Anthias et al., 2012), its insights have been unevenly implemented in policy measures of European countries with growing proportions of immigrants (Lesińska, 2017). This becomes apparent in persistent gaps between official policy objectives and real migration policy implementation in some European countries (Gil Aráujo, 2010; Sánchez-Alonso, 2011).

As seen in Table 1, official migration policies are homogeneous and offer several routes to regular residence in Germany, Italy, and Spain. The latter range from public and humanitarian to private issues, and most are shaped around skilled employment within the knowledge economy. However, due to biases in design or inadequate policy instruments (Sánchez-Alonso, 2011), Italy and Spain stand out with estimated high proportions of undocumented immigrants often being pushed to the margins of social life.

**Table 1:** Overview of migration policy regulations in Germany, Italy, and Spain.

	**Germany**	**Italy**	**Spain**
Migration law	Zuwanderungsgesetz 2005 (Immigration law) Blue Card 2012 (highly-skilled) Skilled Workers Immigration Act 2019	Martelli Law 39/1990 (Regulation of stay) Turco Napolitano Law 1998 (duties to be respected and rights to be granted) Bossi-Fini Law 2002 (Introduction of entry limitations and increase of expulsion decrees) Maroni Law ‘Security Package’ 94/2009 Blue Card 2012 Leg. decree n.108	Ley Orgánica 4/2000 (Immigration law), last reformed RDL 16/2012 Ley de Emprendedores 2013 (Law on Visas for Entrepreneurs)
(Official) Conditions for permanent residence	Depending on migrant status, generally at least 5 years of residence, legal employment + regular income, sufficient knowledge of German language and integration in Germany	Legal and continuous residence for 5 years with legal employment + regular income + sufficient knowledge of Italian language	Legal residence at least 5 continuous years (no language test, no minimum income)
Migration methods	Diverse (Ethnic migration, Work-based permit, Higher education, Marriage, Asylum)	Diverse (Work-based permit, Higher education, Marriage, Asylum)	Diverse (Work based permit, Higher education, Real estate ownership, Marriage, Asylum)
Rights linked to migration policy	Generous (Language support + integration courses Qualification recognition actions)	Meager (Language support + organized locally integration courses)	Meager
N of undocumented migrants	About 180.000–520.000 (0,2–0,6%) Vogel, 2015	About 610.000 (1%) ISPI, 2020	About 825.000 (1,7%) González-Enríquez, 2008 + estimations based on Registro Central de Extranjeros, 2018
Legalization of undocumented migrants	Strict (exceptional leave to remain)	Generous amnesties 1982 until 2020 2002 Amnesty: the largest mass regulation in the history of immigration Colucci, 2018 2009 Amnesty: exclusively for domestic helpers and caregivers	Generous (amnesties and “Settlement Programme”)

*Source: own elaboration*.

Recent studies have argued that residence status insecurity largely shapes access to employment and privileges, placing poorly-educated migrants at a disadvantage (Kofman, 2007; Markova et al., 2019; Sandoz, 2019; Vianello et al., 2019; Lenz, 2020). In other words, unclear perspectives of stay in a host country entail insecure life planning and social inequality, since several temporary residence renewals are practiced in all countries prior to permanent residence. The essence of the countries' policy measures signifies immigrants' responsibility for securing means of subsistence in order to remain. Proof of valid employment contract and language proficiency is often a common prerequisite for a durable/permanent residence or even so-called “legalization” in a destination country. The former is particularly true for Germany that sets value on financial independence and German language skills, subject to approval by relevant authorities. Although Germany has only recently recognized itself as an immigration country with respective legislation (BAMF, 2019), qualification recognition has a sturdy tradition and addresses especially better-educated immigrants (Kogan, 2012). In Italy and Spain, both socioeconomic development and policy measures have long underestimated the need for integration of migrants and focused predominantly on border controls and regulation of immigration. This caused a segmentation of the labor market and development of certain niches with low-quality and precarious jobs absorbing the non-native population (Aretxabala and Gorodetska, 2012). For about 20 years, both Italy and Spain have been characterized by a lack of awareness of this phenomenon, which has become a structural issue. Instead of introducing organic standards capable of regulating migration processes, both countries have launched generalized amnesties, limiting their actions to rejections and expulsions (International Organization for Migration, 2012; Caponio, 2013; Saraceno et al., 2013). In Spain, official amnesties seem to be de facto barely generous in legalizing undocumented immigrants, since denials of applications have been rising within regularization programs (Sabater and Domingo, 2012). Yet, the overall flexibility of Spanish regulations, coupled with high migration outflows, has not caused an increase in irregularity of migrants. Italy, by contrast, has taken its cue from Germany with its “Security Package” (2009–2012), introducing stricter and uniform measures as prerequisites of a permanent residence permit (Caponio, 2013; Stuppini, 2013; Colucci, 2018).

Since a gap between formal migration policies and their factual implementation constrains individual possibilities for employment, reproductive work in domestic service and care activities, mostly falling into the informal economy, represent a professional field in which migrants find employment more easily (Sarti, 2004; Scrinzi, 2004; Barazzetti, 2007; Lutz, 2010; Kofman and Raghuram, 2015; Vogt, 2019). In all three countries, less “useful” foreign workers are more likely to be concentrated in lower-skilled sectors, performing temporary, risky, humiliating, precarious, and less profitable jobs (Ambrosini, 2011; Lenz, 2020). Their constitutive aspects relate to the coexistence of flexibility and subjugation, personalistic relationships and abuse, demand for high productivity and conditions of weakness (Castells, 1989). This condition of vulnerability contributes to producing a framework of economic insecurity, which affects the psychological and emotional dimension of migrant women, and destroys the notion of work in migration as a safe source of income.

### Migrants' Embedding Into Host Countries' Economy

While past literature has discussed migrants' strategies to access employment and examined outcomes for individuals and society, it has somewhat sidelined migrants' management of economic insecurity. Within the latter strand, studies have mostly focused on barriers and coping strategies for finding or remaining at a workplace, frequently low-skilled or informal (Krivonos, 2015; Fangen et al., 2016). This study looks at the junction of employment and unemployment patterns and its dependence on immigrants' values and culturally rooted understandings of insecurity. Are there any alternatives to formal employment to feel economically secure?

The concept of “differentiated embedding” (Ryan et al., 2015; Ryan, 2018; Wessendorf and Phillimore, 2019) can shed light on this by considering diverse dimensions of belonging—micro, meso, and macro. Unlike the static concept of integration which considers migrants' rootedness in a host society in a linear and unidimensional way (Phillimore, 2012), differentiated embedding means attachment to a host society from temporal, spatial and relational perspectives, which implies change of migrants' ties and feeling of belonging over time. Ryan (2018) argues that it is possible to understand different degrees of embeddedness into various domains, such as family, workplace, neighborhood, or personal ties. For instance, a person might be fully engaged with very demanding and time-consuming work, but have no time or opportunity for knowing their neighborhood or even have many friends. Similarly, leaving friends behind might weaken transnational ties over time and result in a gradual dis-embedding from friendly relations and loss of ties to countries of origin. This offers a less linear way of understanding relationships over time. Moreover, depending on life events, the process of embedding might be reversed, as in case of divorce or unemployment.

Ryan (2018) explored patterns of embedding of Polish migrants in London by focusing on the dimensions of family, work, friends circle, and neighborhood, thus primarily tackling the micro and meso areas. She could hardly capture the macro, or institutional, domain associated with embedding, since migration flows from Poland to the UK represent intra-EU movements. While Polish migrants have immediate rights to work and reside in the UK, post-Soviet immigrants originate predominantly from non-EU countries, with an exception of members of Russian diaspora in the Baltic states, and their civil rights differ considerably, depending on their reason for entering the EU. Thus, women's age, country of origin, level of education, and professional qualification might directly affect women's post-migratory life scenarios. Border policies and residence status after arrival in Germany, Italy, or Spain might thus greatly impact the key dimensions of embedding, which adds another layer to the complexity and dynamics of migrants' attachment and belonging in these countries. We therefore expect post-Soviet women to have different levels and patterns of embedding depending on their country of current residence.

## Methodology

### Data Collection Procedures

To explore patterns of economic insecurity in post-Soviet migrant women in Germany, Italy, and Spain, this study conceptually leans on the grounded theory approach (Glaser and Strauss, 1967; Strauss and Corbin, 1990). This is a widely exploratory, data-driven method serving theory generation through the act of collecting and sorting empirical material. The advantage of this approach lies in consideration of several dimensions around the research subject, which helps provide insights and gain more understanding of the investigated issue. The aim is to provide an accurate theoretical explanation of the concept of economic insecurity, shedding light on it by using data from different sources. This includes qualitative interviews and episodes of participative observation carried out both in person and online (ethnographic research online, performed on social media), as well as analysis of judicial documents allowing us to deepen our gaze on migrant biographies from post-Soviet countries, considerably expanding the sample under study.

The grounded theory approach allows scrutinization of the origins of the motives and types of migration from FSU countries to Germany, Italy, and Spain. Secondly, it incorporates the country contexts with their various legal frames, on the one hand, and migrant women's beliefs regarding insecurity, values, life goals, cultural background, and socioeconomic characteristics, on the other. The third dimension is description of actions and activities of post-Soviet women in their host countries, with particular interest in the comprehension of relationships between events, situations, and individual decisions. These three dimensions culminate in consequences, i.e., strategies of migrant women, based on the interplay of the levels of agency—macro, meso, and micro. Apart from highlighting the strategies women develop to cope with economic insecurity, the grounded theory approach contributes to a conceptualization of meanings of insecurity and different patterns of coping, giving grounds for policy implications and recommendations.

**Figure 1:**
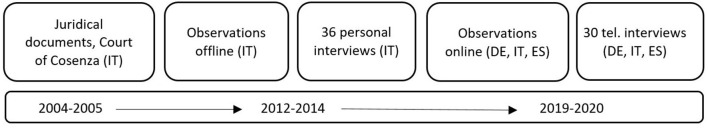
Overview of methodology and data.

Methodologically, this study is built on three pillars of information sources, integrating data from online and offline observations, interviews, and juridical documents. Development of a conceptual codes scheme took place through open coding on each pillar. Codes were discussed by the authors, with special attention paid to interrelations between the concepts through axial coding, as suggested by Strauss and Corbin (1990). The pillars are described below (s. Figure 1).

The first *pillar* of empirical material draws upon a series of participant observation, both offline and online. The former was firstly conducted in Italy during the course of sojourns within Ukrainian families and then during journeys by vans, the so-called *maršrutki*. These are nine-seater minibuses (six for passengers and three for drivers), which have been connecting weekly, for almost 20 years, Italy to Ukraine, transporting women, parcels, and goods of different type. During these journeys from and to Ukraine, open participant observation was conducted with the aim of provoking conversations and narrations starting from questions and curiosities referring to things or situations observed. Traveling on minibuses was one of the most interesting and emotionally-challenging moments of the research for at least two reasons. First, during travel time, a tiny community is formed that reproduces the development of society in a nutshell, represented as it is by men, driving the vehicle, and by migrant women—sometimes accompanied by their children, both Ukrainians and Italians—who spontaneously tell their individual migrant biographies and ways of experiencing the two sides of migration. Within this peculiar space, men posed as transnational voices, positioned as they are within the boundary and contact between the two countries of migration through which they commute.

The observation effort was expended in the exploration of the spaces and their conformation, trying to grasp the difference (and the relevance) between migrant and non-migrant homes and between landscapes and places of departure and landing settings. Interactions were also the subject of attention, especially those relating to ordinary activities, among which we have tried to isolate the most interesting for the research purposes: the relationships of these women with their spouses and their children within home, and, outside, exchanges and behaviors with other migrants, with acquaintances or other people in public places.

Online participant observation data originated from online interactions between post-Soviet migrant women in Germany, Italy, and Spain. Ethnography online helps address the target group digitally and, drawing upon the chosen strategy of (non-)participation as a researcher, observe the groups under investigation, thus gaining valuable information on their meanings, perceptions, and opinions without potential bias of social desirability. Since digital means of communication are pervasive, especially true for social media, online ethnography makes it possible to access very diverse actors. This allows us to not only address the ways women with different social backgrounds experience economic insecurity and how they cope with it, but also obtain rich information on women's age, socioeconomic background, and cultural norms and values.

**Figure 2:**
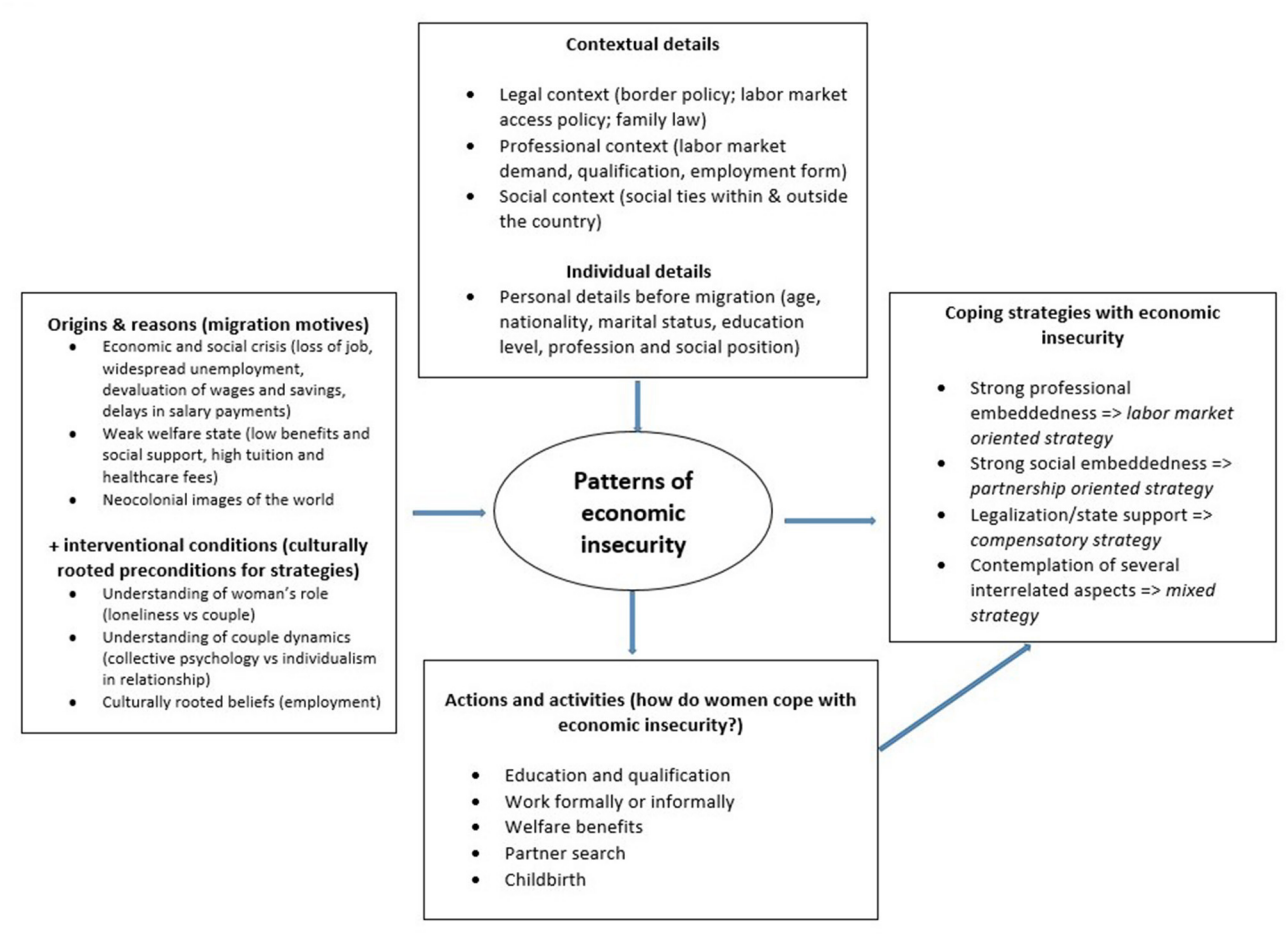
Overview of patterns of coping with insecurity.

Data collection took place via Facebook, the largest global digital community that offers direct interaction between users. There are numerous groups on Facebook that offer digital homes to post-Soviet migrants, where they interact, find friends, discuss difficulties, share experiences, search for help or advice, or simply spend time talking in their native language. Three particular Russian-speaking communities on this social media platform were chosen: they are only for women living in a respective country, amounting to 35–55 thousand users each and representing a space for communication between women on diverse topics. Collecting data was possible, on the one hand, through methods of non-participant observation, non-outing as a researcher in order not to bias the ongoing discussions. At the same time, collection of empirical material was enriched through targeted discussions on women's opinions and attitudes toward their sense of (cultural) belonging, decisions on migration, private issues, such as relationships with European men, etc. Posts in the Russian language in these Facebook communities addressed various questions: why and how women entered their host countries, whether women work out of need or out of self-realization, what obstacles they face when entering partnerships with European men, how unpaid work is divided in their households, etc. Throughout the discussion, it was easy to screen the responses and compile relevant comments. Data gathered from over 20 online discussions resulted in around 1,600 valid comments which were transferred to MaxQDA, broken down to assign meanings and generate pattern codes, with subsequent conceptual coding taking the first step of analysis. Results of further coding and interrelations between the identified concepts are presented in Figure 2 in the Findings section.

The second *pillar* of the empirical data consisted of face-to-face and telephone interviews. In Italy, 36 face-to-face interviews with migrants of Ukrainian origin were conducted both in Italy (province of Cosenza, Calabria) and in their country of origin (in the cities of L'viv, Ternopil', and Ivano-Frankivs'k). Telephone interviews with 30 highly-skilled post-Soviet women in Germany, Italy, and Spain complement the empirical material gained through online observations. These interviews were necessary in order to build a reference category, consisting of well-educated and economically active women who face little economic hardship or extreme situations of insecurity. Respondents were entrepreneurs, scholars, and healthcare workers, exposed to challenges and insecurities regarding their career advancement rather than to illegal or unskilled employment (Gewinner, 2019, 2020). Their experiences demonstrate another dimension of economic insecurity and accomplish the broad spectrum of potential scenarios of coping, with socialization within the similar cultural context of countries of origin being the common denominator for all post-Soviet migrant women.

The *third pillar* of the empirical data is judicial material dating back to 2003, consisting of about 200 phone tappings of telephone conversations between two post-Soviet migrant women, living in the South of Italy, and hundreds of women coming from the FSU to seek job opportunities in the domestic sphere in Italy. These two women acted as intermediaries between job seekers and Italian customers, mostly families. One, of Ukrainian origin, domiciled in a municipality from the province of Cosenza, and the other, of Moldovan origin, resided in a municipality in the Neapolitan hinterland.

The judicial documents, due to their mixed public-private nature, could be classified as a middle ground between “institutional documents” and “personal documents.” This rendered them personal documents with a public-institutional character: “institutional documents,” as material produced by the institutions “regardless of the researcher's action” and “for purposes other than those of social research” (Corbetta, 2003, p. 115–116). We drew upon this material for cognitive reasons, whereas “personal documents” also provide a spontaneous narration of individual experiences and events. Such documents turned out to be particularly interesting because they present unfiltered first-hand material (since the intercepted subjects were completely unaware of the use that would be made of their conversations). They deliver information on the type of work and the conditions in which it is carried out, shedding light on the important elements of the context, especially in reference to the peculiarities of the type of demand and offer. Finally, this material allowed us to arrive at a mapping of the activities carried out by migrant women from the FSU within Italian territory, revealing the dynamics and mechanisms of relationships with their employers, reactions and emotional and psychological consequences—between which lay insecurity and the perception of vulnerability—produced by the migratory parable on migrant life courses.

### Sample Description

Since the aim of the study covered conceptualization of economic insecurity and methods of coping, rather than tracing each woman's personal history, the precise number of study participants, identified through online observations, are not determined. In online discussions that represented a certain discourse, women expressed their opinions multiple times, and the 1,600 comments constitute the actual material based on online observations in the three countries. The diversity of women's attitudes and viewpoints can be observed together with a variety of intersectional characteristics (age, ethnic background) and position in the socioeconomic structure of the country of origin. Women engaged in online communication were aged 20–60, most obtained a qualification prior to migration, yet only some were economically active in Germany, Italy, and Spain.

In Germany, the diversity of women was the strongest and to some extent tied to the motives of migration: romantic reasons (relationship, marriage, or family reunion), repatriation or ethnic migration (Jewish or German origin), as well as (further) education and work. The highly-skilled women in particular had already studied in their country of origin and their migration motive was frequently either further studies (Ph.D., further postgraduate courses, or additional study programs) or economic activity in the host country. The latter incorporates two major scenarios. The first is facilitated particularly by Germany, which forced recruitment of highly-skilled professionals from non-EU countries by launching Blue Card 2012, a temporary residence permit that additionally allows family reunification. Germany is the main profiteer of this EU directive, attracting 84,5% of all highly-qualified professionals, whereas Italy accommodates 1,2% of skilled individuals (BAMF, 2019). Women from Russia and Ukraine each represented one third of all highly-skilled professionals entering Germany (BAMF, 2019). The second scenario incorporates a conspicuous amount of post-Soviet migrant women who pursue research, migrate as health workers, or achieve recognition of their qualifications in order to integrate into the labor market. Highly-skilled women, interviewed by phone, were either employed full-time (scholars, medical doctors) or entrepreneurs in the tourism and hospitality industry. Their average age was about 37 years, they all had at least one higher education degree or Ph.D., and most were in stable partnerships or married.

Among the communities of female foreigners residing in Italy, the post-Soviet migrant women stand out with the high rate of feminisation exceeding 70% (Immigration Statistical Dossier, 2019). They represent almost two thirds of the entire volume of female migrants in Italy: Ukraine (77.6%), after Romania and Albania, retains the leading position in Italian territory, followed by Moldova, Poland, and Russia. This is reflected in interviews with middle-aged women. In the context of countries of origin, these women worked mainly in the education system (one third of the sample is represented by teachers of all levels), in skilled professions (some female engineers and a geologist), and in technical-operational roles (shop assistants, cooks, accountants, and secretaries). In Italy, all found positions in the domestic and care circuit, since job opportunities on Italian territory for post-Soviet female migrants are often limited to the reproductive sphere—domestic work, care of old or disabled people, childcare, or in the domain of entertainment services. Most left children behind—and, in some cases, even grandchildren—for whom they decided to embark on migration. The gradual breakdown of social guarantees represents one of the founding pillars of the Soviet culture and is a key reason pushing these women to leave. Loss of job or increasingly poor salary stimulated women to migrate, reinforced by inflation and the need to support (grand-)children in their university education, property acquisition or renovation, or costly health care.

Post-Soviet women in Spain unite features of their counterparts in Germany and Italy, with migration motives ranging from private and romantic (marriage) to lifestyle migration (climate zone change, remote work in a place of personal choice). A high number of women either accompanied their husbands or traveled to Spain alone and remained illegally in order to avoid returning to their country of origin. Those who came to Spain 15–20 years prior to this study and stayed there illegally could legalize their residential status through amnesties. Similar to Germany, post-Soviet women in Spain in the sample were mostly from Russia and Ukraine and well-educated. However, many faced job scarcity and were forced into either economic inactivity or less-skilled jobs. Women interviewed over the phone were all entrepreneurs in the tourism and hospitality sector, who built up their own business within the holiday apartment sector. They had already stayed for at least 5 years in Spain, all married or in stable partnerships, and most had children.

## Findings

Before discussing the several patterns of coping with economic insecurity, we firstly highlight the meaning of insecurity for post-Soviet migrant women in Germany, Italy, and Spain, as this is the cornerstone in understanding the strategies women develop to overcome post-migratory challenges. Perception of economic insecurity is culture-sensitive and even individualistic, which renders it necessary to fully comprehend how women define it for themselves. This will help understand the women's subsequent coping strategies.

### Meanings of Economic Insecurity

According to the data underlying this study, post-Soviet migrant women's apprehension of economic insecurity is tightly bound to several culturally-rooted concepts that are not obvious at first glance and which represent hidden mechanisms behind consequential coping strategies. First, insecurity is related to the understanding of being a woman. Against the background of their West European counterparts who tend to value independence and freedom, post-Soviet women position themselves as more family-oriented. They are likely to hold views on the necessity of being a mother and official wife in order to be entitled to social benefits or husband's support.

*It is important for a woman to give birth and bring up a child; it is also a kind of self-Fulfillment*.

*The nature of woman is being a mother, taking care of family, making home comfortable. I'm happy being wife and mother and I feel sorry for women who have to work—they don't have any free time and usually look tired*.

Depending on socioeconomic background and gender socialization, they differ in conservative and egalitarian beliefs, yet those attached to more traditional values understand economic insecurity as scarcity of financial resources in present and future, but do not consider (further) investments in education or qualification an option. In this case, marriage would compensate the shortcomings caused by lack of human capital and its loss through the act of migration. Since marriage is deemed one of life's successful scenarios for post-Soviet women (Gewinner, 2019), this fits well the concept of man's responsibility for woman's happiness and security. By contrast, highly-educated women define economic insecurity as temporary employment and the necessity to secure a stable work contract, concerns about losing qualifications, and being forced to work in fields not really matching their expertise or level of competence.

Working as a researcher at a university is the best way to start a family while working part-time. You get a break from home at work and a break from work at home. So with 50% employment you can combine family and work. Another thing is that contracts are usually temporary and you have to constantly worry about extensions, new projects…

…*once I had to make a decision—either leave my habitual environment behind or move to my boyfriend in Germany and start everything from scratch. This was not easy, but I gave the chance to our relationship. My main concern was the job search here, but with my partner's support, I received acknowledgment of my qualification and now work in a hospital*.

Second, understanding of economic insecurity of post-Soviet women in Germany, Italy, and Spain is relational as it is bound to expectations of the host country. This reflects the ingrained neocolonial images and invisible divide between Eastern and Western Europe. In that sense, women anticipate not only better (or any) income, but also better working and life conditions in receiving countries:

*I haven't known much about Spain, but I heard it's very nice, so I enrolled in university studies. Some time later, I started arranging holiday apartments for my acquaintances, and at some point I understood that I really like it, so now I'm trying to further establish my own business*.

Additionally, they hope to support relatives left behind and offer a better life to their (future) children by enabling them to move and reuniting with them. Hence, women are ready to take any job if they cannot redeem their qualifications, even if it downgrades their social status, for the sake of their children's future education and opportunity to live in a more secure and prosperous country. Clear patterns of inequality within the group of migrant women is observable here, unveiling the hierarchies in whiteness based on citizenship. Those who possess EU citizenship (Romania, Bulgaria, or Baltic countries) have the privilege of legally remaining in Italy or Spain, and especially in Germany due to intra-European open borders regulation. Even women from Ukraine enjoy the advantage of the visa-free policy regime, being able to enter the EU and remain in a chosen country for a period of 3 months. The opportunity of longer stay is linked to employment conditions in the host country and potential social obligations in the country of origin.

Third, economic insecurity is bound to the uncertainty of the return and of readapting oneself to the reality of countries of origin, made of economic and working difficulties, corruption and lack of social opportunities. This is linked to women's changed perception of the context of departure:

Ladies, I need your advice—what should I do? I moved to my boyfriend in Italy, but after some time, he completely changed. I'm trying not to annoy him, but he is aggressive and I'm afraid of him. I don't have any income in Italy, I stay alone at home the whole day and don't know what to do. I thought of returning back to my country, but have nothing left there and actually I don't want to go back to this hell…

The prospect of legalization in Italy and Spain or obtaining a long/permanent residence permit in Germany greatly influences the decision whether or not to leave the country. In this cost-benefit analysis, retaining access to the host country outweighs other considerations and persuades women to stay at the cost of poor jobs and separation from close relatives.

### Patterns of Coping With Economic Insecurity

We find four incisive strategies implemented by women to escape the trap of economic insecurity (s. Figure 2). On the one hand, they are related to the social spheres in which women are predominantly embedded, but on the other hand, these strategies are guided by the norms with which post-Soviet women were socialized. The success of a respective strategy depends on the circumstances of interaction between the norms and the migration venues.

#### Labor Market-Oriented Strategy

Professional development strategy is organized around the intention to counteract deskilling and maximize chances for positive quality of life after the migration episode. Post-Soviet women who follow this strategy have common features that unite them into a certain distinguished profile. These women usually originate from well-educated families, known as *intelligentsia* (Bassin and Kelly, 2012; Perotto, 2014; Savikovskaia, 2017; Malyutina, 2020), where one or both parents have an academic background. They often self-initiate migration and largely rely on themselves in pursuing administrative procedures, communicating with potential employers and seeking useful information that might help them swiftly adapt to new life conditions. Their life values fit especially the German context coined by job diligence and work ethics:

*I get along very well here. After many years of hard work, I finally have a fascinating job, very good income and can afford many things*.

*I have never experienced job rejections, I could always choose from the best job offers. Yes, it's been a long way, but now I can say, it was worth it. I can't imagine my life without work*.

The social reproduction within the *labor market-oriented strategy* is remarkable. Human capital, accumulated through socialization within academic parental family, coupled with internalized normality of participation in paid employment and ideas of gender equality, stretches beyond country borders and represents a powerful mechanism of protection against deskilling, loss of identity, and depression. Women who experience temporary unemployment due to their newcomer status (e.g., as accompaniers of husbands), loss of qualification because of poor transferability to the labor market of the host country, or protracted process of communication with the foreigners' office, seek escape from this personal crisis. In Germany, they approach state employment agencies and seek German language courses, integration courses, job preparation courses, or continued education. Their persistence and efforts sometimes exceed the competence of local civil servants who act as gatekeepers and meet decisions over women's further occupational life courses:

I was surprised when I realized that I was better informed about my case than the lady in charge of my documents at the employment office…

In Italy and Spain, women comprehensively inform themselves online using official webpages and social media, seeking legal consultations to navigate the less regulated immigration system and fulfill their projections to keep or even enhance their own social status. Yet, since Italian and Spanish policies are still rooted in state nationalism, few qualifications can be officially recognized. This is why younger women enroll in universities, which is deemed inappropriate by those in their late 30s as it is perceived to be too late to study. This gives the latter a feeling of not being needed and bereft of a substantial part of their lives. A common way to overcome this consists of devaluing paid work *per se* and convincing others that housework is as hard work as regular employment:

*As a matter of fact, “work” originates from the word “slave”* [in Russian]. *When a woman is supported by her husband, she belongs to herself. She is the one who decides about her time, does what she wants to do and what she wants to leave for later*.

Work is not a woman's job! Making the house nice and clean, taking care of children, cooking is work as well. Look at women who work—they are just enslaved!

A quite different scenario develops when post-Soviet women deliberately downgrade the value of their own qualifications and agree to perform any job for the sake of leaving their country of origin. Several aspects impact crucially on the outcomes of this. First, age is a decisive element in what kind of job these women are offered. Based on the translated juridical documents and information from the interviews, we find that the largest part of the migratory flow to Italy was constituted by women between 35–40 and 50–55 years. We refer to a situation existing about 20 years ago (among the interviewed women, the first had arrived between 1997 and 1998: so if these women had not returned, they currently would be between about 55 and 70 years of age, except for a few 30-year-olds who would be about 45 now). An age-related neat dividing line influences working opportunities of younger women (30–40) and women over 40: hierarchization occurs here not only on the basis of age, but also of physical appearance, which determines the type of work the women will do. The younger group, which is, however, a minority and whose arrival in Italy goes back to some years before they were 30, has generally experienced a more diversified working career. Initially introduced to the outdoor services (restaurants, pizzerias, and pubs), they have ended up babysitting or, in some cases, working in local factories.

Secondly, current residence country is another crucial aspect within the *labor market-oriented strategy*. There is a difference between women already residing in host countries but who have restricted access to better segments of the labor market, which is more the case in Germany, and those who migrate deliberately for work, mostly in Italy and Spain. Particularly women who have not put down roots in the countries of destination enter them to start a job on a co-residential basis and remain in that position in the receiving context for the whole period of stay, seeking to maximize their earnings and better benefit their children and remaining families. This is especially true for women from Ukraine and post-socialist EU countries. Women married or cohabiting with their partners in destination countries are more likely to opt for a live-out model, which is the case particularly for women from Russia. The latter model includes heterogeneous life constellations of women: some work when the possibility arises; some study and, if they can, do some work, and some could not find anything but cleaning and caregiving activities. Job opportunities for individuals without (recognized) qualifications, therefore, are almost always channeled toward the same working environment, outside of which it is not easy to find anything else.

In summary, taking responsibility over their own life and professional embedding is inherent to migrant women from post-Soviet countries. Depending on their socioeconomic resources and educational background, women are inclined to participate in paid employment or establish their own business in Germany, Italy, and Spain in order to facilitate better life conditions for themselves and their close relatives, the latter usually being vulnerable due to young or elder age. In many cases, women compensate shortcomings of the marriage market, as well as uncertainty and discriminatory practices faced in the countries of origin. However, host countries' national policies and their openness to migrants tend to “channel” these women in an interplay of individual and country conditions, and produces several patterns of participation in the labor market.

#### Partnership-Oriented Strategy

When the first strategy does not succeed as expected, marriage assumes a greater meaning. Marriage (more desired than a stable love relationship) becomes a way to purge constricting social and professional dynamics; it represents the possibility of escaping economic insecurity and a method of sharing family responsibilities with a reliable partner. Partner search fulfills two purposes: not only can it help gain permanent residence status, but it can also accomplish a second life success scenario, since family has the highest cultural value for most post-Soviet women. For the realization of this scenario, women are ready to accept the role of a housekeeper and withdraw themselves from the labor market because available jobs are likely be precarious and of low quality, thus hardly contributing to women's self-realization.

A man is someone who takes responsibility for his family and acts as breadwinner. I believe a real man is someone who is attentive and makes a woman feel good, makes gifts, provides help, supports her financially…

*To me, being married means being confident in future, being secure. A good wife is there to motivate her husband to earn money because he has a family to take care of*.

Well, if a man does not want to marry, then he's just playing with me. It means he does not want to share his life with me, does not regard me as a right choice and is not serious enough about our relationship. A man would never let the right woman go!

The image of a caring and supportive man borders on an ideal type of partner, whose nationality is less important. This is especially true for post-Soviet women in Germany with men of European or FSU origin. In Italy and Spain, mixed marriage is likely to be an option, consisting of a groom from a destination country and a bride from the post-Soviet space. In addition to economic and professional insecurity, this could also be due to the image that these women build about “the West” and the life that it offers, which embodies the idea of well-being they have always craved. This makes them “want the country and the partner” (Beck and Beck-Gernsheim, 2012, p. 115). One of the reasons behind this choice could be the very common belief among post-Soviet women that men from the FSU lack reliability and the ability to take care of their families and companions emotionally and materially. In the representations of many women, post-Soviet men are unrelated to the management of family affairs and unfaithful, in addition to exceeding, in some cases, in violent and self-injurious behavior (referencing in particular the widespread problem of alcoholism).

*What I like about German men is that they are clearly more family-oriented, sharing household responsibilities is a natural thing apart from paid work… they are usually good fathers and take care of their children. If I see men on a playground, these are most likely Germans*.

My ex-husband was 5 years older than me; my Italian partner is 10 years older, and it is as if he were wiser. My ex is as if he had remained with a thoughtless head. He was not aware he had a family. My current partner is more responsible, more used to thinking of the family, sounder, makes economies, tries not to spend money: he uses his head!

It's difficult to find a suitable Spanish partner, or maybe I'm doing something wrong. I read stories of other girls in this group how happy they are about their Spanish partners, but I wasn't that lucky so far. Where do they find them?!

According to the available data, a substantial proportion of post-Soviet migrant women opt for marriage or cohabitation with a German, Italian, or Spanish man. However, there is a thin line between wanting the partner and wanting the country. Some studies have shown a significant reduction of occupational disadvantage for migrant women in a mixed couple, due to their deeper embeddedness into wider and more diversified networks (Ambrosini, 2011; Ballarino and Panichella, 2015). The option of the marriage bond, therefore, translates itself into a process of release from the conditions of vulnerability and social and occupational constraint, which represents the possibility of escape from situations of deprivation.

*He says he is in love with me. Me, in love?… Maybe it's more affection than love… but he knows this. When I go to Ukraine I miss him. I am grateful to him for all he has done for me and for the children, because he always does whatever is necessary*.

We identify several scenarios of *partnership-oriented strategy*. While migrant women with transferable, “desired” qualifications are more likely to enter a partnership in the destination country, women with poor professional prospects find partners within the (care) working environment (possibly a relative of a client) or seek a foreign partner still residing in the country of origin. Within the first scenario, women have the bargaining power and are more likely to meet a partner based on sincerity and real affection. In the second, women relieve themselves of the burden of heavy and constricting work, sanctioning the regaining of their autonomy and social position as first-rate citizens, making the transition from “servant” to “lady.” In the third case, women decide to elude the futile job search and instead, seek to embed into social relations of a host country first, only then seeking professional opportunities. The latter scenario is often fatal for women's personal and professional development. Searching for a partner as a bridge to the host country entails serious disadvantage for post-Soviet migrant women, since the idealization of Europe and expectations of a better life render them unaware of potential dangers and loss of identities and the life they had in their country of origin. This often results in loneliness, helplessness, and even domestic violence.

It is worth looking into the meanings of femininity deployed by migrant women for partner search. Regarding Italian and Spanish, but especially German, women as too emancipated, less attractive and not caring about their appearance, many post-Soviet women consider themselves much more feminine, tender, fragile, weak and thus worth caring about. They believe this would awaken a protective instinct in men, making the latter feel needed and appreciated. Moreover, women try to find a man because in their understanding, a single woman is a lonely and unhappy woman. This cultural value means that a woman *has* to have a partner as this is how her value is measured. Making oneself attractive (nails, hair, lips, lashes, nice clothes, etc.) signals “taking care of oneself” and finding a man.

*I invest my time in skincare, make-up, nice clothes, hairdresser, nail artist—just to always look attractive and be especially interesting for a man. So this is kind of self-explanatory that he should compensate my investments by paying the bill when we go out. There is hardly something more disgusting than a man who wants to divide a bill*.

*Women who try to be independent just envy those who get everything paid by men! How miserable one should be, how humiliating it is for a woman to pay the bill! Real women allow the man to pay for her, this is an art in itself that we haven't lost despite feminism*.

Considering that German, Italian, and Spanish partners are more often older or “mature” men in second marriages, marriage translates into a dignified and useful accommodation for both parties. On the one hand, a match with a traditional woman benefits men who tend to a return to a more traditional (way of doing) family, with a woman who, in addition to embodying a peculiar type of beauty, is also more care-oriented and less afflicted by career concerns (Salvino, 2017). On the other hand, a woman looks for a safe point of reference to finally feel free from many loads and worries, just by activating her charm and being a “real woman.” Frequently, women are aware of their disadvantage in the labor market of their host country, and seek to fulfill the second strategy to success, marriage. This is where cultural norms of migrant women positively interact with a certain niche of the marriage market in the host country. This scenario turns to danger when a man expects traditional division of labor within the partnership, or is not attractive in the local marriage market. Entering a relationship based on online “catalogs of brides” is often fraught not only with professional degradation, but also physical and mental abuse and violence against women. In this case, women are left alone without means of subsistence, with broken hopes and needing to start a new life from scratch.

#### Compensatory Strategy

A wish to stay in the host country and secure better life conditions results in a hybrid form of coping with economic insecurity, which is mostly pronounced in Germany and is a consequence of the failed *partnership-oriented strategy*. Women with no or poor qualifications, and sometimes bad partnership experience, rely on the rather generous state benefits that largely substitute a partner. Although not very common, this constellation is not new to migrant women, since many either grew up without a father or witnessed a lived practice of the Soviet gender contract, where women had the state rather than a man in the role of a partner (Temkina and Rotkirch, 2002; Antoshchuk and Gewinner, 2020). To be entitled to these benefits, women hasten onto motherhood, entitling them to support from (ex-)partner and the state, and also anchors women's civil right to enter and reside in the country. In this case, either a post-Soviet man with permanent residence permit or respective citizenship or native European man becomes a bridge, or a stepping stone, to the country of a woman's choice. Discussions in Italian and Spanish, but especially in German, Russian-speaking online communities demonstrate that these are often young women of childbearing age who materialize their migration projects at the cost of others.

When I worked in a store while studying, I had a colleague, a young girl who was already a mother. She regularly tried to “help me” with her experience—saying she would give me contacts of right people who would arrange my social benefits, connect me with employers who pay gray salary so that I'm still eligible for state support, and so on. She genuinely couldn't understand why I always refused!

When I moved to my Spanish partner, there not many job possibilities for me, so I concentrated on our relationship. Actually, my husband didn't want children, but I happened to get pregnant… Now our relationship doesn't seem to work anymore. My husband wants to get rid of me after 2 years, what can I do to stay in Spain? I heard I might be entitled to some benefits?

Becoming a mother is one of the highest normative values in the system of meanings for post-Soviet women. In their understanding, woman's nature rests on motherhood and care, and these women seek to fulfill their role believing that a new country would open more possibilities for that. They largely rely on others—a man as breadwinner and supporter or a state, and often hardly consider the chance of their being left alone with child(ren) without means of subsistence. This cultural ideal thus turns to a hidden mechanism of coping with insecurity in the host country. Women's disillusionment with partners lies in the lack of possibility to fulfill the cultural norm, especially when partners do not conform to women's “mentality.” Even if partnerships are coined by oppression of women or violence, women prefer to retain them until they are eligible for state benefits and permanent residence in the host country.

*I always wanted to have my own small nice family, but my husband seems to care more about his children from the first marriage than about our daughter! He spends too much time with his other children, constantly gives his ex money, and she thinks she can call him anytime and talk to him. While I'm just serving them when his children are in our house. I don't know how to reduce his contact with his previous family, this is really annoying*.

*I met a German man through one dating platform; he visited me a couple of times in the country I live in. It was very nice, he said he liked me. When I told him that I was pregnant, he said, he was not ready for this so soon and ignored me since then. I believe he has to take responsibility for his child, so I'm looking for ways to claim my right for his support and residence in Germany*.

Against the background of embedding into the system of (state or individual) external support, women's misuse of men, on the one hand, and civil rights, on the other hand, is overt. This circumstance can be hardly categorized as incidental, since women develop strategies to overcome policy requirements toward participation in the labor market. Romantic relationships in this case often do not last longer than the period of time needed to obtain a permanent residence permit in a respective country. Besides, women following the *compensatory strategy* are usually well-informed about the ways to feign sickness or other obstacles in order not to work. Their cost-benefit analysis leads them to elude poor and insecure jobs that barely offer more income than state benefits. Instead, women following this strategy are likely to seek a new partner to take responsibility for them and their children. Having a formal job is thus more insecure than being married or having a relationship with a man who supports them.

#### Mixed Strategy

The *mixed strategy* implies either double and simultaneous embeddedness into several life domains, or mixed situations that contemplate several interrelated aspects. The most prominent examples are a mixture of social and professional embeddedness, but there can also be social and state or state with professional scenarios. For instance, highly-skilled women in Germany can exemplify strong attachments to both work and family when they seek tenured positions or a promotion.

*We are an unconventional family in every sense – we both work in different cities, we constantly agree on plans to take care of our son and consider it normal. My husband and I try to spend more time together, then it's an island of relaxation for us*.

*Our working day is divided into two shifts: in the morning my husband takes care of the children, makes them breakfast and takes them to school. I am already at work and come home in the evening. He always cooks, and after dinner it's my shift—I take care of the children, we play together, I put them to bed, I read aloud to them*.

Spain represents an interesting case of *mixed strategy* of coping with economic insecurity, where legal regulations and individual pursuits toward paid employment pay off and convey a clear improvement of socioeconomic status after state amnesties. Here, as in Italy, many migrant women remain in the country and search for legal employment contracts in order to be eligible for a permanent residence permit/citizenship. They feel encouraged to work officially, since they are then entitled to some social benefits, such as child support or the payment of pensions, for contributions accrued in the country of destination, once they return home. This example demonstrates how social status and contradictory social mobility can change several times through the very episode of migration with initial loss of social status, and then upward mobility through occasional governmental actions. This strategy, however, requires temporal and monetary resources on the part of migrant women, since the process of legalization can take many years and human capital is challenged to remain competitive and attractive to employers.

Another peculiar case of *mixed strategy* can be found in Italy and consists in spending one's free time in cultural, social, or religious associations, both indigenous and ethnic, in order to feel more fulfilled and integrated. This strategy ranges from singing or conducting choirs to practicing massages in local churches, teaching one's own language (or Russian language) to the host population or second-generation children, to intellectual engagement in ethnic or local newspapers (Vianello, 2014), and the creation of specific associations that protect the rights of migrants, offering them different services. Here, in addition to temporal and monetary resources, a certain degree of rootedness in the community is also required, in which the migrant women must have developed a more or less extensive network of contacts.

## Discussion and Conclusion

This study investigated the meanings of economic insecurity from the perspective of post-Soviet women residing in Germany, Italy, and Spain, thus giving voices to migrants and their experiences in several European countries comparatively. Defined through the perceptions of the research participants, it discloses strategies women use to improve their living conditions in receiving countries in relation to work and private life. Building upon grounded theory, the methodological approach integrated several sources of information based on conceptual codes elaborated in the course of analysis. This made it possible to disentangle the hidden mechanisms underlying coping strategies, largely being implicit cultural norms and notions of femininity deeply rooted in migrants' mindsets. Moreover, this methodological procedure demonstrated how different cultural values are being activated and how they interact with the migration venues depending on the context. Consideration of the macro dimension particularly adds to the debate and shows women's embeddedness into it as a substitute to men. We found that migration policy plays an important role for obtaining the right to stay in the host country in terms of life planning and confidence in the future. The countries' context in terms of legislative frames and labor market structure enriched the results, helping distinguish and contrast strategies used within each context and in relation to the country of origin.

At the micro level, the four identified coping strategies are largely shaped by age, qualification, residential status, and cultural norms of women, all interacting with the migration venue in specific ways and resulting in different outcomes. If education and degree are “useful” for the host country, this facilitates women's embedding into the labor market, which leads to social status attainment and extended opportunities for partnership formation. Marriage is a strategy implemented by migrant women in order to overcome the vulnerability that distinguishes them as migrants and women, achieving the goal of economic emancipation and promoting social mobility, so that the migration path can be successful, almost taking on the value of a ransom. Our data indicate that for each niche of marriage market in host countries, there is a sort of “offer” made by post-Soviet migrant women based on the set of characteristics they bring with them, which creates certain lovescapes (Cvajner and Sciortino, 2021). Childbirth and reliance on partner and/or state benefits shapes the third strategy, which contributes to women's vulnerability and social exclusion.

Our results show that in Germany, insecurity is shaped by institutional frame conditions and filtering of migrants in skilled and unskilled jobs, which has its roots in the legal prescriptions. The interplay of macro and micro features produces multiple strategies deployed by post-Soviet women, and these strategies are most diverse here. The latter range from professional fulfillment to dependence on external actors, such as the state or partner. In Italy, insecurity has much to do with immigration policies and irregular residence closely related to the migrant and care work status, which implies consequences such as sudden loss of employment and a home to live in. The most prominent coping strategy is, thus, the partnership-oriented one, even often for well-educated women. Nevertheless, with the consolidation of migratory flows from the East, alongside the *partnership-oriented strategy*, the possibility of moving toward more sustainable forms of mobility is emerging for an increasing number of migrants. The itinerant circular mobility of relay migration prevails, which, alternating periods of stay at home with work stays in Italy, is characterized as an effective resilience strategy capable of limiting the social and emotional costs of these peculiar transnational female migrations (Salvino, 2018). Spain has common features with both Germany and Italy, where migrant women are likely to be channeled to both skilled and unskilled positions in the labor market depending on their initial migratory motives, and where women tend to rely on their partners to handle insecurity. The common denominator of all strategies is often the permanent stay aspiration in the host country and citizenship, with only a low proportion of women remaining mobile and having several migration episodes in their lives.

Insecurity means not so much lack of financial resources as the paucity of institutionalized belonging to the host country in form of permanent residence permit or citizenship. Although coping strategies with economic insecurity of post-Soviet migrant women in the three countries are tightly linked to economic activity, they are simultaneously implicitly rooted in individual understandings of the role of woman and femininity. Women actively apply their “erotic capital” (Hakim, 2010, 2011) to gain security through their partners, which sometimes turns out to be a dangerous investment. Thus, formal employment is not always seen as secure way of life; instead, being married and supported by a man is a frequently quoted way to feel safe and secure.

The findings show that, although the effective way of overcoming insecurities is embeddedness in the formal labor market, since all described strategies are organized around professional fulfillment, gender equality becomes a rather flexible term. Post-Soviet women, socialized under ideas of equality and women's obligatory employment, bow to traditional gender role models when migration to Europe is promised, being ready to transform from “mistress” to “maid” (Young, 2001) for the sake of better life conditions for themselves and their children. This causes a certain cultural backlash to neotraditionalism in Germany, Italy, and Spain, supporting the conservative part of the male population.

In this respect, economic activity as self-realization is a strong empowerment instrument that pays off for better-skilled post-Soviet women abroad regardless of the support of traditional values. In Germany, highly-skilled women in good labor market positions do not consider a man an economic resource, but they perceive it natural for a man to be the breadwinner. Less skilled women or those without recognized certificates implicitly perceive a man as a source of income. This points at the commonality of the cultural value of a man taking care of the family. This notion is more strongly pronounced with increasing age of women, which contributes to the debate on intersectionality.

We believe that these strategies apply to most migrant women in the EU and elsewhere, yet this should be a subject for further research. Moreover, interaction of imported cultural norms with host countries represents another research avenue. Learning more about women's strategies of coping with insecurity can help reduce women's vulnerability in host countries and empower women. Therefore, we deem communication of (un)successful migration scenarios online and offline helpful, to aid women in avoiding falling victim to violence and exploitation in host countries, assistance remaining an act of charity and individual issue, with the state removing itself from the scenario.

## Data Availability Statement

The datasets presented in this article are not readily available because the data are generated from several sources of information and need to be edited in order to guarantee the full anonymisation. Requests to access the datasets should be directed to irina.gewinner@uni.lu.

## Ethics Statement

Ethical review and approval was not required for the study on human participants in accordance with the local legislation and institutional requirements. Written informed consent for participation was not required for this study in accordance with the national legislation and the institutional requirements.

## Author Contributions

All authors listed have made a substantial, direct and intellectual contribution to the work, and approved it for publication.

## Conflict of Interest

The authors declare that the research was conducted in the absence of any commercial or financial relationships that could be construed as a potential conflict of interest.
