# Supplementary figures and images for: Understanding Patterns of Economic Insecurity for Post-Soviet Migrant Women in Europe
Source: Front Sociol. 2021 Apr 15;6:614713. doi: 10.3389/fsoc.2021.614713 (PMC8083980; doi:10.3389/fsoc.2021.614713)

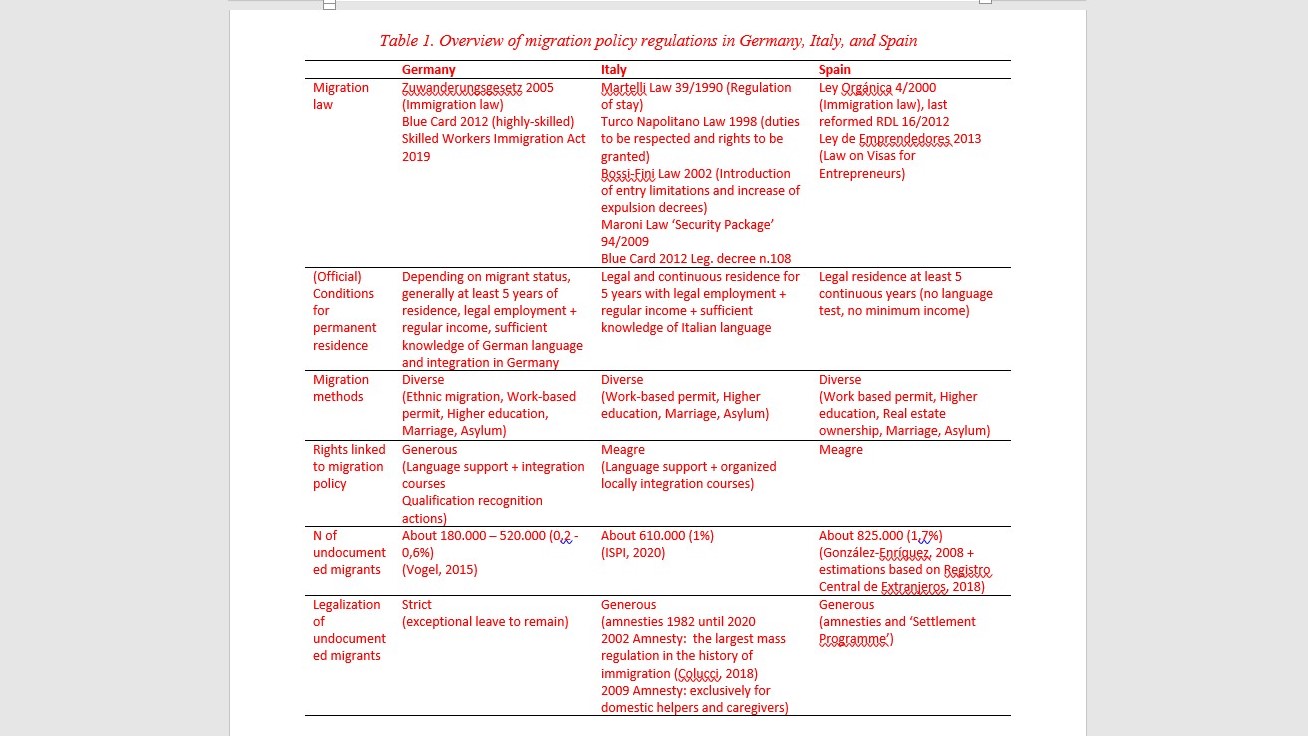

Supplement: Supplementary file 1 [file Image_1.JPEG]
